# Breakdown of *Arabidopsis thaliana* thioredoxins and glutaredoxins based on electrostatic similarity–Leads to common and unique interaction partners and functions

**DOI:** 10.1371/journal.pone.0291272

**Published:** 2023-09-11

**Authors:** Yana Bodnar, Manuela Gellert, Faruq Mohammed Hossain, Christopher Horst Lillig

**Affiliations:** 1 Institute for Medical Biochemistry and Molecular Biology, University Medicine Greifswald, Greifswald, Germany; 2 Institute for Physics, University of Greifswald, Greifswald, Germany; North-Eastern Hill University, INDIA

## Abstract

The reversible reduction and oxidation of protein thiols was first described as mechanism to control light/dark-dependent metabolic regulation in photosynthetic organisms. Today, it is recognized as an essential mechanism of regulation and signal transduction in all kingdoms of life. Proteins of the thioredoxin (Trx) family, Trxs and glutaredoxins (Grxs) in particular, catalyze thiol-disulfide exchange reactions and are vital players in the operation of thiol switches. Various Trx and Grx isoforms are present in all compartments of the cell. These proteins have a rather broad but at the same time distinct substrate specificity. Understanding the molecular basis of their target specificity is central to the understanding of physiological and pathological redox signaling. Electrostatic complementarity of the redoxins with their target proteins has been proposed as a major reason. Here, we analyzed the electrostatic similarity of all *Arabidopsis thaliana* Trxs, Grxs, and proteins containing such domains. Clustering of the redoxins based on this comparison suggests overlapping and also distant target specificities and thus functions of the different sub-classes including all Trx isoforms as well as the three classes of Grxs, *i*.*e*. CxxC-, CGFS-, and CC-type Grxs. Our analysis also provides a rationale for the tuned substrate specificities of both the ferredoxin- and NADPH-dependent Trx reductases.

## Introduction

Redox modifications of cysteinyl side chains are a vital part of numerous signal transduction pathways, in photosynthetic organisms these mechanisms play a vital role, *e*.*g*. in light-dark adaptation [1–3]. Redox modifications of protein thiols such as disulfide formation and reduction are catalyzed by members of the Trx family of proteins, *i*.*e*. Trxs and Grxs [3–7]. This group of proteins share a common structural motif, the Trx fold. Cysteinyl residues in their active sites are the basis of their redox activity [8]. The proteins of this family catalyze the oxidation and reduction of disulfides in target proteins, including glutathionylation-deglutathionylation of proteins. Trx family proteins are encoded in essentially all genomes and they were likely already present in the last universal common ancestor of all life forms an earth [9]. Trxs and Grxs are present in all compartments of eukaryotic cells, *e*.*g*. the cytosol, ER, mitochondria, nucleus, and plastids–often in multiple isoforms [10]. Most Trxs and Grxs have a broad, but distinct substrate specificity. Understanding the molecular basis of their target specificity is key to their physiological functions and for the understanding of redox regulation in general. This molecular basis is the focus of this work.

Trxs are efficient catalysts of thiol-disulfide exchange reactions and the trans-nitrosylation of cysteinyl side chains [11, 12]. During their reaction cycle, Trxs form a disulfide in their Cys-Gly-Pro-Cys active site. This disulfide is reduced by thioredoxin reductases (TrxRs). Photosynthetic organisms contain two types of TrxRs. First, the ferredoxin-dependent FTRs that couple the photosynthetic electron chain directly to redox regulation [13] and, second, the NADPH-dependent NTRs that function, among others, in stress defense [14]. Grxs are divided into three classes [4, 15, 16]. The first, also named class-I or CxxC-type Grxs share a Cys-Pro-Tyr-Cys consensus active site motif and catalyze thiol-disulfide exchange reactions, often including glutathione (GSH). Oxidized Grxs are reduced by two molecules of GSH. The resulting GSH disulfide is reduced by GSH reductases at the expense of NADPH. The second subclass of the Grxs, named class-II or CGFS-type Grxs, with a consensus Cys-Gly-Phe-Ser active site, do not catalyze thiol-disulfide exchange reactions. Instead, they function in the regulation of iron metabolism or in the transfer of iron-sulfur centers [17–19]. Two alternative loop structures preceding the active sites and different modes of GSH-binding resulting thereof are the basis for the fundamentally different functions of these two Grx classes [20, 21]. The proteins of the third class are restricted to land plants, they were named ROXYs or CC-type Grxs. These proteins function in the regulation of TGA family transcription factors. So far, neither redox, nor FeS transfer activity of these proteins was described that could be linked to their physiological functions. In most species, and in plants in particular, various Trx family protein isoforms were described and characterized in the same compartment, prompting questions on both overlapping and distinct functions. Proteomic studies screening for interaction partners and target proteins, also summarized in this work, imply a high degree of substrate specificity for the individual isoforms.

The basis for this specificity has long been a mystery. Previous hypotheses addressed the thermodynamics of the reaction, foremost the nucleophilicity of the more N-terminal active site thiol and the resulting differences in their standard redox potentials [22, 23]. Also favorable entropic contributions as the major recognition force controlling the specificity of these protein-protein interactions were suggested [24]. Based on the analysis of *Escherichia coli* phosphoadenylyl sulfate reductase, we proposed that different electrostatic properties of the redoxins govern their target specificity and reactivity [25, 26]. A comprehensive analyses of Trx family proteins from human and various other species confirmed that primary and tertiary structure similarity do not correlate to the target specificity of the proteins. Instead, a number of examples clearly demonstrated the importance of electrostatic similarity for their target specificity [27]. In this work, we have compared all redoxins encoded in the genome of the model plant *Arabidopsis thaliana*. Our results emphasize the importance of the electrostatic properties and suggest some unexpected close functional ties between all classes of redoxins.

## Methods and procedures

### Structures and molecular modeling

Available structures were obtained form the protein data bank (https://www.rcsb.org), all pdb entries are specified accordingly. The Swiss Model web server was used to perform molecular modeling [28–31]. Based on the quality assessment provided, *i*.*e*. the lowest QMEAN with no major outlier in the global or local quality estimate of Cβ, all atom, solvation, or torsion, the final model was chosen from structures modeled with different templates that displayed the highest sequence identity with the target protein. S1 Table in S1 File summarizes the individual template structures used as well as the QMEAN values.

### Sequence and structure comparison

Protein sequences were obtained from the uniprot resources. Alignments of the primary structures and generation of the corresponding distance trees were performed using the CLC sequence viewer (Qiagen bioinformatics, Hilden, Germany) and Clustal omega [32]. PDBeFold server (v2.59, https://www.ebi.ac.uk/msd-srv/ssm/cgi-bin/ssmserver) was used for alignment of the three dimensional structures, the structure-based multiple sequence alignments were then prepared with ClustalW2-”Simple Phylogeny”-tool (https://www.ebi.ac.uk/Tools/phylogeny/) with default settings. Corresponding distance trees, based on these primary structure and 3D structure alignments, were generated with the iTOL (https://itol.embl.de/) [33] viewer.

### Electrostatic calculations

Alignment of the structures in the PDB files in the desired orientation was performed using UCSF chimera. The proteins electrostatic properties were computed from the pdbs as follows: using pdb2pqr with the amber force field, missing atoms were reconstructed, hydrogens added, atomic charges and radii assigned [34]. Calculation of the electrostatic parameters was performed using the Adaptive Poisson-Boltzmann Solver (APBS) [35] within the vmd (visual molecular dynamics) software package [36]. The parameters were set to the following values: 150 mM mobile ions, a temperature of 298.15 K, and a solvent dielectric constant of 78.54. From these data, images were rendered representing the secondary structures including the N-terminal active site cysteinyl residues facing forwards, the electrostatic potential mapped to the proteins water accessible surface from -4 (red) to 4 *k*·T·e^-1^ (blue), and the isosurfaces of the electrostatic potential at -1 (red) and 1 *k*·T·e^-1^ (blue). Applying ImageMagick a summary picture was generated. All steps following the 3D alignment were automatized, the scripts and a graphical interface are available at: https://github.com/WillyBruhn/MutComp.

### Electrostatic distances and clustering

Using the Gromov-Wasserstein-distance approach described before (27), we compared the 3D isofsurfaces of both the negative and positive electrostatic potential. Solving this problem, is NP-hard as the objective function is not convex, however, in polynomial time three lower bounds for the Gromov-Wasserstein-distance can be calculated. We limited the sample to 100 points randomly distributed on the isosurfaces and calculate the lower bound for them. Following an 500 times repetition, the values obtained were summarized in form of a histogram. This comparison was performed pairwise for all proteins. The earth-mover’s-distance was used [37] to calculate the similarity between the histograms, the unweighted pair group method with arithmetic mean (UPGMA) was used for hierarchical clustering, see [27]. This method yields the mean distance between all points from the new cluster to all points of another cluster and the results were displayed in form of a dendrogram. The software source code is available at: https://github.com/BerensF/ComparingProteins.

### Interactome data and comparison

Interactome data were retrieved from the IntAct database [38] (as of Sept. 2018) as well as the BioGRID resources [39] (Vers. 3.4, as of Sept. 2018). The uniprot resources (https://www.uniprot.org) was used to perform ID mapping and all entries are listed with their unique UniprotKB ID. Additional resources for interactions, *e*.*g*. from some dedicated publications, were included, when available. S1 Table in S1 File (interactome) contain all identified entries. Computation of the matrix of common interactions and the Venn diagrams was performed using R (https://www.r-project.org) from RStudio (https://www.rstudio.com).

## Results and discussion

In plants, Trx family proteins were originally named and classified according to their specificity for certain redox-regulated substrates. For Trxs, for instance: F-type (fructose-1,6-bisphophate phosphatase), H-type, and M-type (malate dehydrogenase). Subsequently, redoxins with similar primary structures were named accordingly with added consecutive numbers in order of their discovery. The complexity of the plant redox systems, summarized in Table 1, is astonishing. Given that a substantial number of them function in redox regulation and all of them will function through direct protein-protein interactions, the molecular determinants of their specificity for distinct and common target proteins are central to the understanding of their regulatory functions. We have proposed that complementary electrostatic patterns control this specificity [25–27]. The aim of this study is to provide a thorough comparison between clustering and classification according to primary structure, 3-D structure, and electrostatic similarity. Further more, we discuss the implication of the clustering of electrostatic properties for the functional classification of plant Trx family proteins and potential overlaps in target proteins. The electrostatic characteristics and similarities were computed as outlined before [27]. Table 1 also lists functions of the proteins as described in the literature. Potentially interacting proteins were collected from primary literature as well as interaction databases, *i*.*e*. IntAct and BioGRID (see methods section and S1 File). For ten proteins (GrxC5, GrxS14, GrxS16, TrxF2, TrxH1, TrxM1, TrxM2, TrxO1, TrxO2, and Trl33), 3-D structures were obtained from the protein data base (pdb). The remaining structures were predicted by homology modeling.

**Table 1:** Redoxins and redoxin domains encoded in the *Arabidopsis thaliana* genome.

*Arabidopsis thaliana* glutaredoxins					
protein name	ID	active site	pdb	functions	Interactome (no. of interactions)
GrxC1	Q8L8T2	CGYC		Fe/S (redox sensor), pollinization [40],	30
GrxC2	Q9FNE2	CPFC		redundant to GrxC1 [40]	-
GrxC3	Q9FVX1	CPFC		DHA reduction, (de)-glutationylation [41]	1
GrxC4	Q8LFQ6	CPFC		similar to GrxC3 [41]	1
GrxC5	Q8GWS0	CSYC	3rhb, 3rhc	Fe/S [42] (redox sensor)	2
GrxC6	Q8L9S3	CCMC			1
GrxC7	Q96305	CCMC		petal, anther development [43, 44]	-
GrxC8	Q8LF89	CCMC		floral development [44]	8
GrxC9	Q9SGP6	CCMC		defense gene expression [45, 46], SA and JA signalling [47]	9
GrxC10	Q29PZ1	CCMC			1
GrxC11	Q9LYC6	CCMC			1
GrxC12	O82254	CCMC			1
GrxC13	O82255	CCLC		hyponastic growth, regulation of TGA1 and 4 [48], nitrogen starvation signalling [49]	1
GrxC14	Q9LYC5	CCLC		hyponastic growth, regulation of TGA1/4 [48], nitrogen starvation signalling [49]	1
GrxS1	Q9SA68	CCMS			1
GrxS2	Q8L8Z8	CCMS			1
GrxS3	O23421	CCMS		down-regulated under high density [50], up-regulated by nitrate [51]	10
GrxS4	O23419	CCMS		down-regulated under high density [50], up-regulated by nitrate [51]	-
GrxS5	O23420	CCMS		down-regulated under high density [50], up-regulated by nitrate [51]	1
GrxS6	Q9LYC8	CCMS			2
GrxS7	Q6NLU2	CCMS		down-regulated under high density [50], up-regulated by nitrate [51]	-
GrxS8	O23417	CCMS		down-regulated under high density [50], primary nitrate response [52]	-
GrxS9	O04341	CCMS			5
GrxS10	Q9LIF1	CCMS			1
GrxS11	Q9M9Y9	CCLS		nitrogen starvation signalling [49]	1
GrxS12	Q8LBS4	CSYS		(de-)glutathionylation [53]	5
GrxS13	Q84TF4	CCLG		protection against photooxidative stress [54], nitrogen starvation signalling [1]	2
GrxS14	Q84Y95	CGFS	3ipz, 2mma	Fe/S transfer [18], control of vegetative growth, chlorophyll content [55]	4
GrxS15	Q8LBK6	CGFS		Fe/S transfer [56]	16
GrxS16	Q8H7F6	CGFS	2lwf	Fe/S transfer [18], ontrol of vegetative growth, chlorophyll content[55]	7
GrxS17	Q9ZPH2	3·CGFS		Fe/S transfer [57, 58]	28
*Arabidopsis thaliana* thioredoxins					
Trxf1	Q9XFH8	CGPC		redox regulation [59], seedling establishment [60], oxidation of Rubisco activase [61]	-
Trxf2	Q9XFH9	CGPC	7c2b	seedling establishment [60], oxidation of Rubisco activase [61]	-
Trxh1	P29448	CGPC	1xfl		47
Trxh2	Q38879	CGPC			-
Trxh3	Q42403	CPPC			73
Trxh4	Q39239	CPPC			1
Trxh5	Q39241	CPPC		defense genes expression [45], plant immunity [62]	80
Trxh7	Q9XIF4	CGPC			97
Trxh8	Q9CAS1	CGPC		stress defence [63]	6
Trxh9	Q9C9Y6	CGPC			5
Trxh10	Q9LXZ8	CVPC			-
Trxm1	O48737	CGPC	7c65		2
Trxm2	Q9SEU8	CGPC	7c3f		4
Trxm3	Q9SEU7	CGPC			-
Trxm4	Q9SEU6	CGPC		redox regulation [59, 64]	6
Trxo1	O64764	CGPC	6g61	redox regulation [65]	-
Trxo2	Q93VQ9	CGPC			-
Trxx	Q8LD49	CGPC		seedling establishment [60]	2
Trxy1	Q6NPF9	CGPC	7bzk		14
Trxy2	Q8L7S9	CGPC			72
CITRX / Trxz	Q9M7X9	CGPC		chloroplast development [66], adapter protein [67]	2
Nrx1	O80763	2·CGPC		catalase protection [68], redox regulation [69]	1
Nrx3	Q8VZQ0	CRPC		redox regulation [69]	-
F4HPP4	F4HPP4	CGPC			-
TR164 / HCF164	O23166	CEVC		cytochrome b(6)f biogenesis [70]	1
Trl11 / Lilium1	O64654	CGGC			-
Trl22 / Lilium2	Q8LCT3	CASC		fructose-1,6-bisphosphate oxidation, may function in NPQ regulation, reduction of Prxs in light-to-dark transition [61]	-
Trl12 / Lilium3	Q9XFI1	CGGC			-
Trl13 / Lilium4	O22779	CGGC			1
Trl21 / Lilium5	Q8LEK4	CGSC	6lyx, 6lyw	redox regulation [71], FBPase oxidation, may function in NPQ regulation, reduction of Prxs in light-to-dark transition [61]	-
Trl4 / Lilium6	Q9C5C5	CGSC			-
Trl33	Q8LCH9	CGVC			-
CXXS1	Q8LDI5	CIPS		disulfide isomerase activity [72]	-
CXXS2	Q8GXV2	CLPS		disulfide isomerase activity [72]	-
Trl31 / WCRK1	Q9FG36	CRKC		Oxidation of ATP synthase CF1-y [61]	-

### Grxs and Trxs in *Arabidopsis thaliana*

The *A*. *thaliana* genome encodes 31 Grxs or Grx domain-containing proteins and 37 Trxs or Trx domain-containing proteins (see Table 1). Unfortunately, only for ten of these proteins experimentally determined structures were available. Homology modeling of the others, however, yielded structure models with reliable quality parameters (see S1 Table in S1 File). The wealth of several 100 template structures for Trx family proteins allows the prediction of valid model structures in most cases.

**Fig 1:**
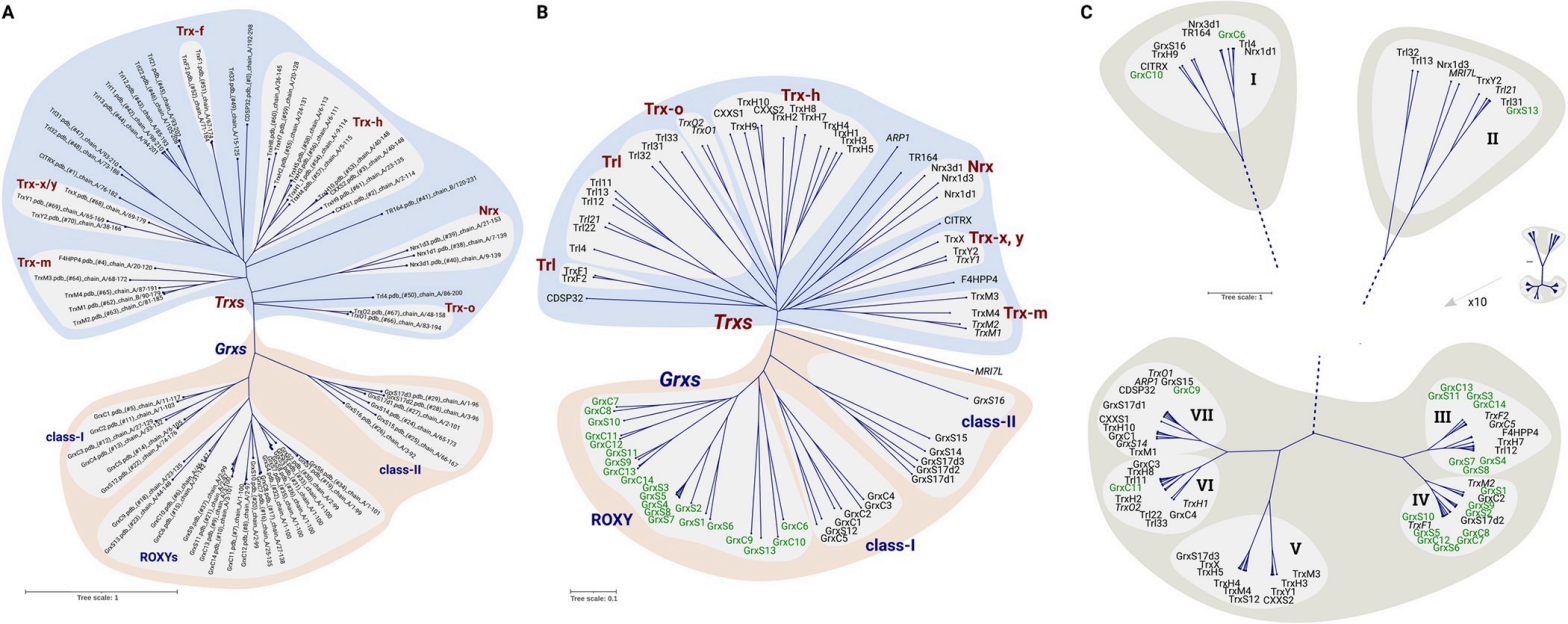
Clustering of *Arabidopsis thaliana* redoxins. (A) Phylogram based on primary structure comparison, computed by Clustal Omega and CLC sequence viewer. (B) Similarity tree based on the similarity of the 3D structures extracted from the pdb and generated by homology modeling; the tree was computed using PDBeFold server (https://www.ebi.ac.uk/msd-srv/ssm/cgi-bin/ssmserver) and the iTOL (https://itol.embl.de/) (C) The electrostatic similarity of the whole proteins was computed as outlined in the methods section; the tree was generated using ‘R’, the tree representation was done with iTOL (https://itol.embl.de/). The protein abbreviations highlighted in green correspond to the ‘ROXY’ classes in A and B.

**Fig 2:**
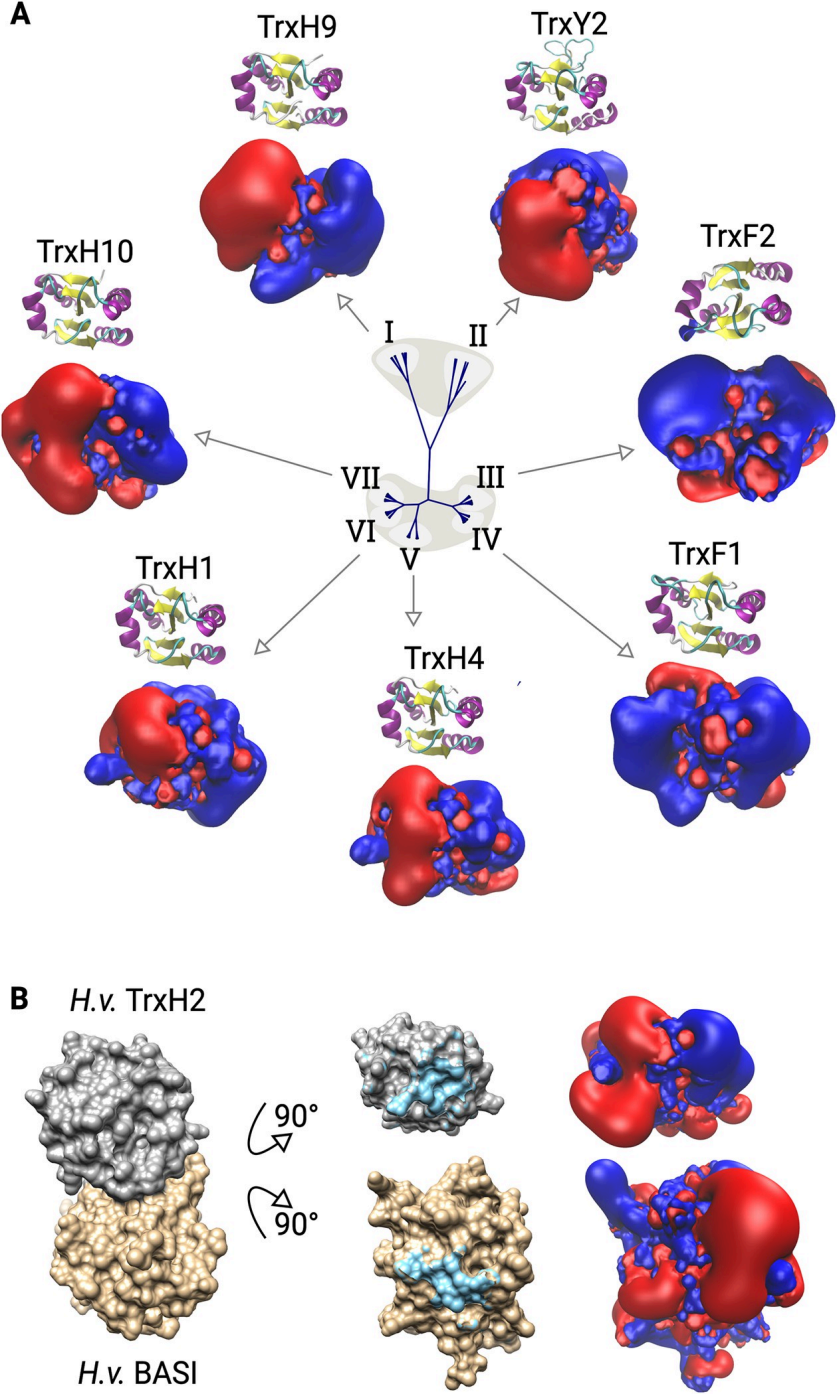
Electrostatic features of the active site contact areas of exemplary redoxins from the seven electrostatic groups of *Arabidopsis thaliana* redoxins. (A) Representative redoxins for each group. Depicted are the cartoon representations of the redoxins with helices in purple, sheets in yellow and the N-terminal CxxC cysteinyl residues in ball-and-stick model. and the electrostatic potential isosurfaces at + and—1 *k*·T·e^-1^, blue: positive, red negative potential. (B) Interaction between barley (*Hordeum vulgare*, *H*.*v*.) TrxH2 and barley alpha-amylase/subtilisin inhibitor protein (BASI), pdb entry: 2iwt. The protein complex was opened (middle) to expose the interaction surfaces highlighted in cyan. The electrostatic potential isosurfaces of the interaction surfaces are shown at + and—1 *k*·T·e^-1^. The orientation of TrxH2 in (B) is the same as one for the other Trxs in (A).

Next, we compared the proteins based on their primary and tertiary structures as well as their electrostatic similarity. Both the clustering according to primary and 3D structures (see Fig 1A and 1B) yielded very similar results. The proteins fall into the previously established classes, *i*.*e*. for the Grxs the CxxC-/class-I-type, the CGFS-/class-II-type, and the CC-/ ROXY-/class-III-type, for the Trxs the Trx-f, Trx-h, Trx-m, Trx-o, Trx-x/y, Trx-like (Trl), and nucleoredoxin (Nrx) groups. The comparison of the proteins’ electrostatic features (Fig 1C, see also Fig 2 and S1 Fig in S1 File), yielded a completely different picture, not in accordance with the current classification systems. The proteins fall into three major groups with in total seven subgroups (Fig 1C, I-VII). All of these groups contain proteins from the Trxs and Grxs families and different sub-classes within these families. Fig 2A displays the electrostatic properties of the protein-protein interaction surfaces of representative members of each of these seven groups (for all details, see S1 Fig in S1 File). Five of these groups (I, II, V, VI, and VII) show a split charge distribution, in this view negative charge on the left and positive on the right, with various unique features. Two of the groups (II and IV) display a predominantly positive potential on this surface with a more neutral area surrounding the N-terminal active site thiol. For comparison, we added an analysis of the only experimentally solved complex between a plant redoxin and a target protein, *i*.*e*. barley TrxH2 with barley alpha-amylase/subtilisin inhibitor protein (BASI) [73] (Fig 2B). This complex highlights the electrostatic complementary of the interaction surfaces, as described for other Trx-complexes before [25, 74]. It also illustrate that the electrostatic differences between the seven groups (Fig 2A) must have a profound impact on their interaction profile.

**Fig 3:**
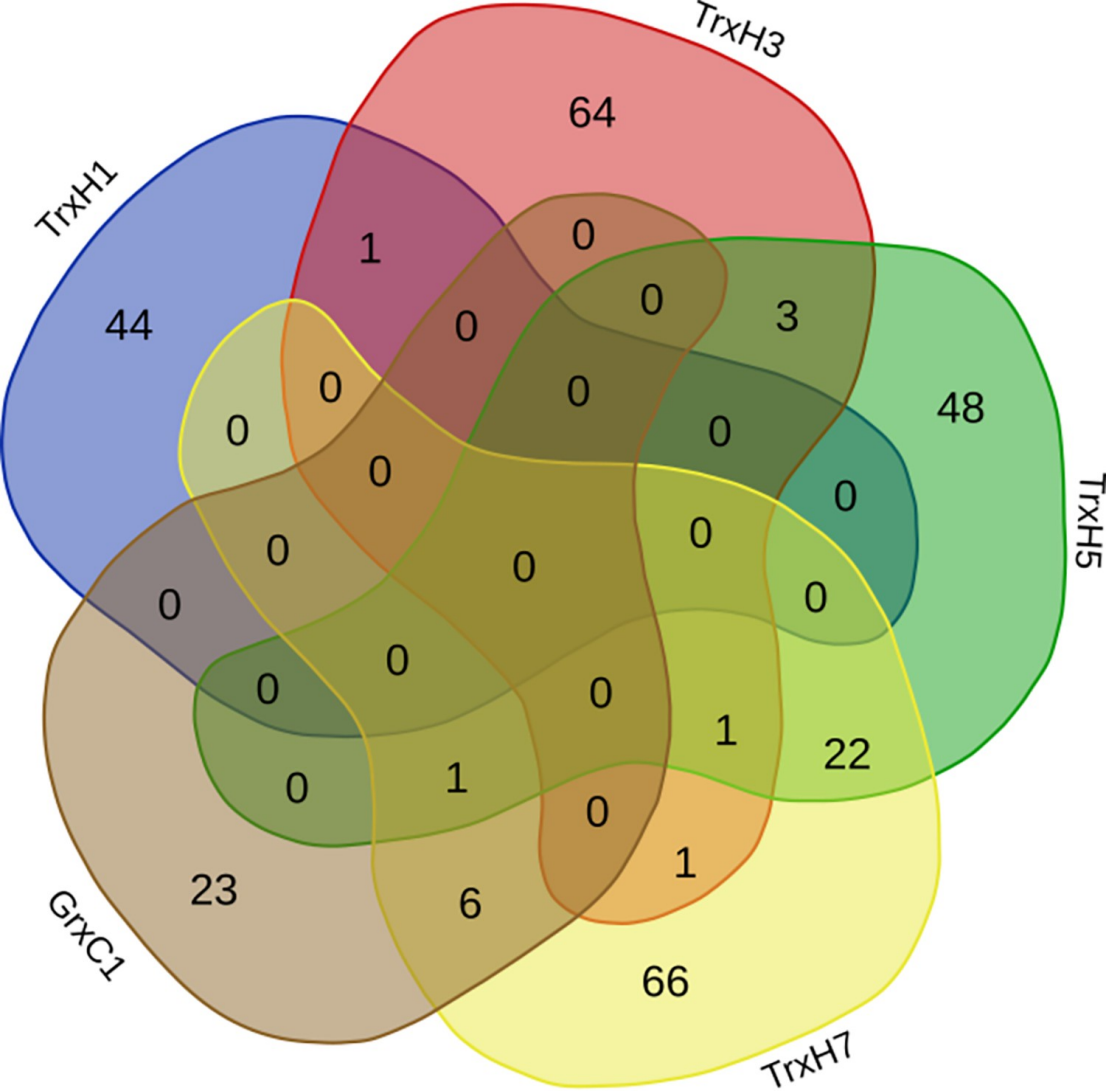
Venn diagram of the potential overlapping interaction partners of the indicated redoxins. The potential or suggested interaction partners are listed in the S1 Table in S1 File and were received from the literature and various interaction data bases.

The data on potential interaction partners of plant redoxins published or deposited in the databases (see S1 Table) was limited. For the following redoxins a substantial number of potential interaction partners could be identified: GrxC1 30 unique interactions, TrxH1 45, TrxH3 70, TrxH5 75, and TrxH7 97. From the total of 317 entries, 280 were only reported as interaction partner for one of these redoxins. The highest overlap (see the Venn diagram in Fig 3) was detected between TrxH5 and TrxH7 (n = 24), the second highest between GrxC1 and TrxH7 (n = 7), and the third highest between TrxH3 and TrxH5 (n = 5). Of course, the data basis used for this comparison (literature and databases) was neither representative, nor obtained in a systematic or comparable way. All of the proteins mentioned before are located in the third major group (II-VII) of our tree (Fig 1), in favor of some common interaction partners.

The CC-type/ROXY Grxs physically and genetically interact with different members of the TGA transcription factor family, however the nature of these interactions (redox modifications or sole binding) and their interactions with potential other target proteins were mentioned as “one of the challenges in the future” in a previous review article [16]. In both primary and tertiary structure clustering, this Grx class-III subfamily form a homogeneous group (Fig 1A and 1B). With respect to the electrostatic clustering, these proteins are spread over all six of the seven clusters with a concentration around clusters III and IV (green labels in Fig 1C). This may indicate that theses CC-type/ROXY Grxs interact with specific sets of different target proteins, with some overlapping functions, for instance of GrxC11, GrxS4, GrxS7, and GrxS8 (see cluster ‘V’ in Fig 1C). The closely related GrxC7 and C8 (ROXY1 and 2) both interact with the transcription factors PAN, TGA9, and TGA10 (summarized in ref. [16]), see S1 Table). This is also consistent with their predicted similarity in electrostatic features (see cluster ‘IV’ in Fig 1C and S1 Fig in S1 File). The high similarity in the predicted electrostatic characteristics between the different CC-type/ROXY Grxs with classical Cys-X-X-Cys-type Trxs and Grxs may indicate that proteins from all major groups share some interaction partners; a hypothesis that remains to be proven.

### Electrostatic similarity of redoxins from photosynthetic species compared to redoxins from other species

**Fig 4:**
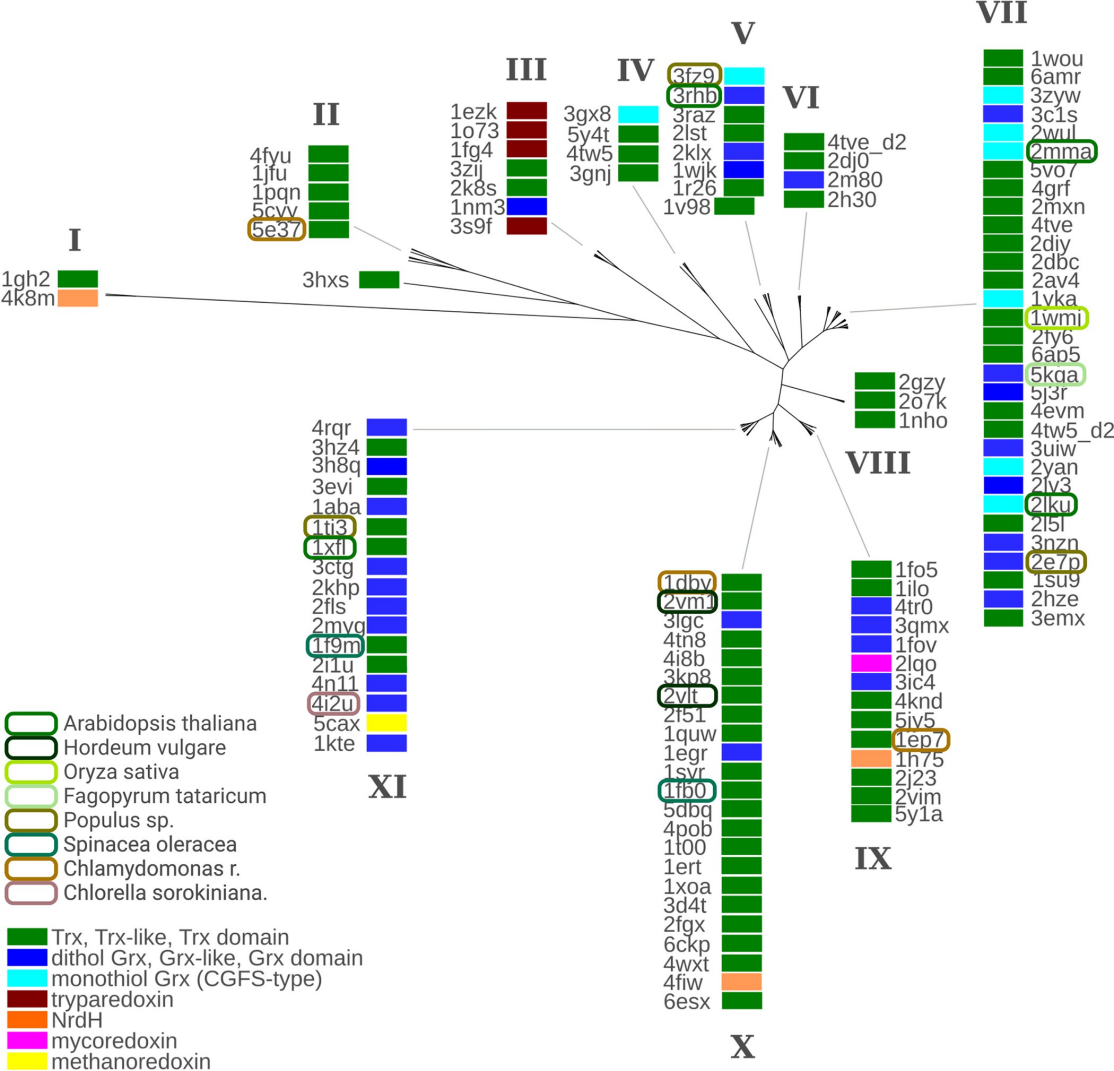
Hierarchical clustering of all redoxins with experimentally determined structures in the pdb. The electrostatic similarity of the whole proteins was computed as outlined in the methods section. All redoxins from photosynthetic organisms were highlighted. The color codes were included in the figure. Modified from reference [27].

Previously, we have reported the comparison of the electrostatic characteristics of all experimentally solved redoxin structures [27]. Based on these data, we have now focused on a comparison of the redoxins from photosynthetic organisms with the redoxins from various other species. We excluded mutated proteins, extracted the redoxin domains if required, excluded other molecules in the pdb files, and focused on one representative structure when multiple entries were available in the pdb structure. We have also excluded all proteins reported to function in oxidative protein folding as well as peroxidases such as peroxiredoxins and GSH peroxidases. The electrostatic similarity tree based on this data set separates into eleven branches, marked as I-XI in Fig 4. Most of these branches include structures from both the Trx and Grx subfamilies. These branches do not correspond to branches of trees generated by comparing primary or tertiary structure similarity [27]. The redoxins from the photosynthetic organisms, as for all other branches of life, do not form separate groups. We speculate that electrostatic similarity corresponds to target specificity, as reported for the model target *E*. *coli* PAPS reductase [25]. This, in turn, implies (electrostatically) similar targets for some redoxins across species barriers. The cytosolic H-type Trxs (1xfl, 1ep7, 2vm1, 2vlt, 1wmj, 1ti3) for instance, are spread over branches VII-XI, the two plastidal M-type Trxs (1dby, 1fb0) are located in branch X. Noteworthy, this branch contains also a number of confirmed electron donors for ribonucleotide reductase from *E*. *coli* (*E*. *coli* Grx1, 1egr; *E*. *coli* Trx1, 1xoa; and human Trx1, 1ert) as well as two Trxs from the H-type (*Hordeum vulgare* TrxH1 and H2, 2vm1 and 2vlt, respectively).

**Fig 5:**
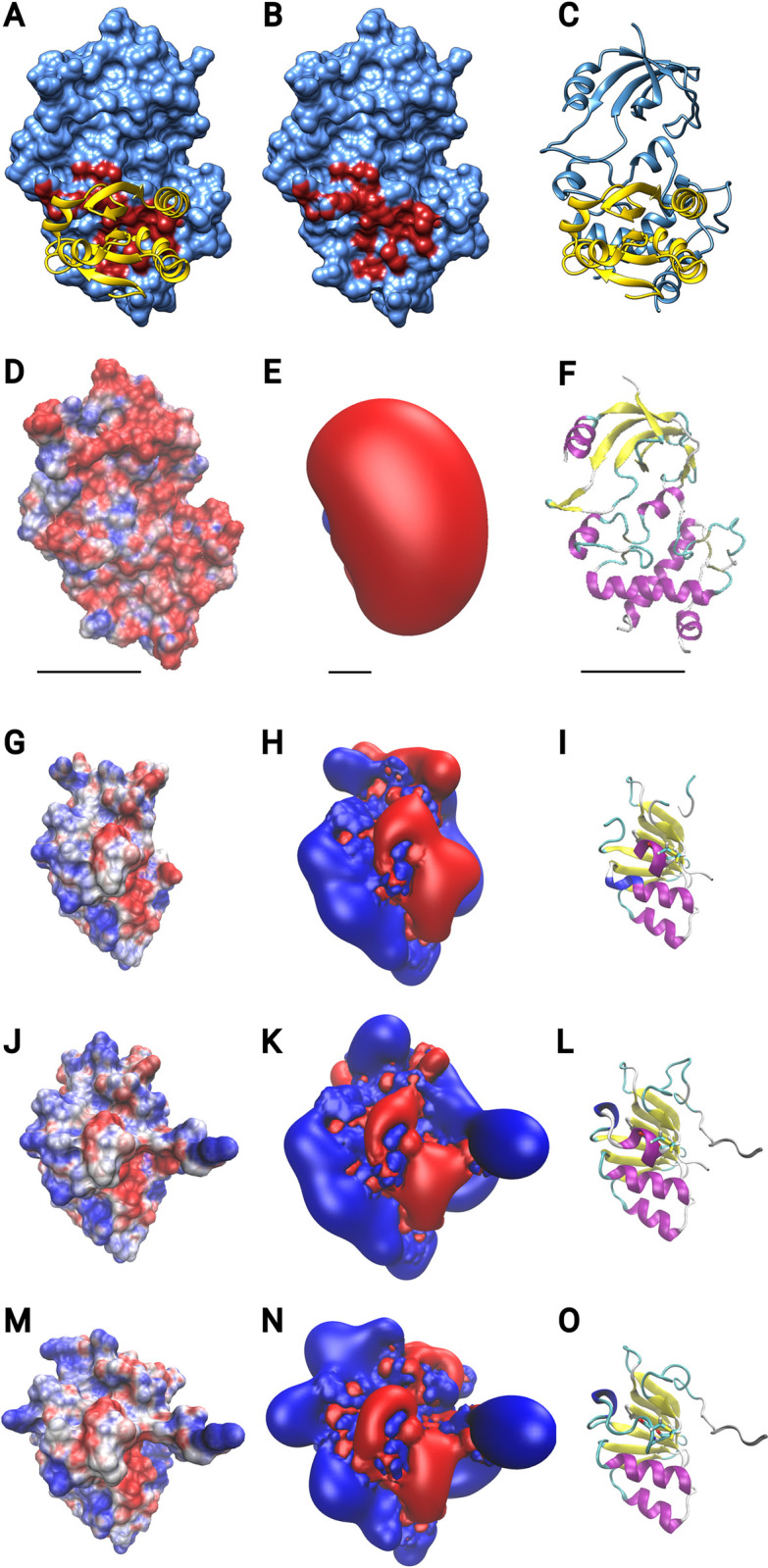
Electrostatic features of the active site contact areas of the thioredoxin reductases from *Arabidopsis thaliana*. (A-C) the contact surface of FTR with TrxF2 (pdb 7c2b) computed with UCSF Chimera. (D-F) the electrostatic features of FTR from 7c2b. The image of the electrostatic isosurface (E) was scaled down by a factor of 5 for better visibility, lines represent the scale. (G-I) electrostatic features of NTR2 domain containing the active site cysteinyl residues analyzed based on pdb entry 1vdc (the FAD domain was removed as explained in the maintext).(J-K): electrostatic features of NTR1. (M-O) electrostatic features of NTRC. NTR1 and NTRC were modeled and based in this structure and analyzed accordingly. The electrostatic potential isosurfaces are shown at +/- 1 k·T·e-1. The electrostatic potential mapped to the water-accessible surface of the proteins at +/- 4 k·T·e-1. Blue: positive, red negative potential.

Plant genomes encode a particularly high number of Trx-family proteins. In general, the number of redoxins, together with the genome size in general, increased during the evolution of more complex organisms. However, the human genome encodes ‘only’ 35, the E. coli genome seven Trx family proteins. The high number of Trx-family proteins in plants must reflect the importance of redox regulation and cellular redox homeostasis in plant biology. Four functions stand out in particular. First, chloroplast and photosynthesis functions, redoxins are essential for maintaining the redox state of photosynthetic enzymes and regulating the light reactions [75]. Second, the regulatory functions of the CC-/ROXY-/class-III-type Grxs that are unique to plants [76], 21 of the 66 Arabidopsis redoxins belong to this group (Table 1). Third, the wide verity of environmental challenges that plants are exposed to, such as temperature variations or droughts. And fourth, specific functions in distinct developmental stages, for instance during seed germination [77]. As outlined above, many plant redoxins show high similarity in their electrostatic properties to their counterparts from other domains of life (Fig 5). The redoxins from the electrostatic similarity groups III and IV (see Figs 1 and 2), however, do not resemble Trx-family proteins from other organisms. These two closely related groups include 81% of the CC-/ROXY-/class-III-type Grxs, and the two F-type Trxs, all of which are unique to plants. Taken together, our results suggest that plants evolved their high variety of Trx-family proteins to fine tune specific functions, similar to all higher life forms, and to cope with unique new targets and regulatory needs.

## Thioredoxin reductases

Unlike other organisms, plants contain two types of Trx reductases. The NTRs include the cytosolic and mitochondrial NTR1 and 2 (also named NTRA and NTRB) [14] and the plastidal NTRC that, unlike all other TrxRs, contains its own C-terminal Trx domain [78]. To better understand their reactivity with the various Trx isoforms, we also analyzed the electrostatic properties of *A*. *thaliana* FTR, NTR1, NTR2, and NTRC (excluding its Trx domain, see Fig 5). The structure of *A*. *thaliana* FTR from FTR-TrxF2 complex published in [79] (pdb 7c2b) was used for comparison. NTRs must undergo substantial conformational changes during their reaction cycle. In all experimentally determined structures, the two cysteinyl residues that reduce the Trx substrates in the dithiol-disulfide exchange reactions face the FAD domain for electron transfer form NADPH. When reducing Trxs, this domain must open up to interact with the Trx. We therefore focused on the surrounding of the reactive cysteinyl residues. The structure of NTR2 [80, 81] served as template for the modeling of NTR1 and NTR3. Our results show some major differences between the two reductase classes. First, the FTRs show a particular large interaction surface with the Trxs of about 756 Å^2^. This is considerably larger than for any other TrxR including the bacterial and human enzymes with around 560 and 450 Å^2^, respectively [82, 83]. The protruding geometry of the active site thiols of the NTRs (Fig 5) suggest a smaller interactions surface for these as well. Second, the electrostatic properties of both classes differ significantly. While the NTRs, including NTRC, exhibit the division of potentials in larger negative and positive potentials with the active site right in between them, the FTR displays a very negative potential facing the Trx interaction surface (Fig 5). When comparing for complementarity, this indicates that NTRs should interact preferably with Trxs that also contain their active sites at the border between a larger negative and positive potential. This is true for most Trxs [25, 27]. For the *Arabidopsis* Trxs (see Fig 2A) this is the case for the Trxs of the clusters I, II, V, VI, and VII. While FTR may preferentially interact with Trxs of clusters III and IV, such as TrxF1 and F2 (Fig 2A). This suggests, in agreement with a previous report [84], higher efficiencies of FTR with the F-type Trxs. Under competitive conditions, these should be the preferred substrates over the M-type TrxM1 (cluster VII), as well as TrxM3 and TrxM4 (both cluster V) implying a hierarchical organization of light-dependent redox regulation in the chloroplast. Noteworthy, TrxM2 is most similar to TrxF1 (both cluster IV).

## Conclusions

Here, we have applied a mathematical model developed for the automated clustering of proteins, in our case members of the Trx family of proteins, according to their electrostatic similarity. The analysis of the plant redoxin structures clearly demonstrates that primary and tertiary structure similarity do not correlate to electrostatic similarity and presumably function, see [25]. The least understood group, the CC-/ROXY-/class-III-type Grxs, for instance, contains various members with high electrostatic similarity to Trxs F1 and F2 (Fig 1C). The clustering according to electrostatic properties represents a model to understand both the overlapping and distinct target specificity of the plant redoxins in their different cellular compartments, they also provide clear clues for the necessity for multiple TrxRs within a given compartment.

## Supporting information

S1 Table Contains the spreadsheet summarizing the interactions partners for the *Arabidopsis thaliana* redoxins. (XLSX)

S1 File Contains S1 Fig (Electrostatic features of the active site contact areas of the redoxins from *Arabidopsis thaliana*), S1 Table (3D structures analyzed in this work), and additional references. (PDF)

## References

[1] Buchanan BB, Balmer Y. Redox regulation: a broadening horizon. Annu Rev Plant Biol. 2005;56: 187–220. doi: 10.1146/annurev.arplant.56.032604.144246 15862094

[2] Buchanan BB, Luan S. Redox regulation in the chloroplast thylakoid lumen: a new frontier in photosynthesis research. J Exp Bot. 2005;56: 1439–1447. doi: 10.1093/jxb/eri158 15851415

[3] Cejudo FJ, Ojeda V, Delgado-Requerey V, González M, Pérez-Ruiz JM. Chloroplast Redox Regulatory Mechanisms in Plant Adaptation to Light and Darkness. Front Plant Sci. 2019;10: 380. doi: 10.3389/fpls.2019.00380 31019520 PMC6458286

[4] Rouhier N, Gelhaye E, Jacquot J-P. Plant glutaredoxins: still mysterious reducing systems. Cell Mol Life Sci CMLS. 2004;61: 1266–1277. doi: 10.1007/s00018-004-3410-y 15170506 PMC11138834

[5] Lillig CH. Glutaredoxin systems. Biochim Biophys Acta. 2008;1780: 1304–1317. doi: 10.1016/j.bbagen.2008.06.003 18621099

[6] Montrichard F, Alkhalfioui F, Yano H, Vensel WH, Hurkman WJ, Buchanan BB. Thioredoxin targets in plants: the first 30 years. J Proteomics. 2009;72: 452–474. doi: 10.1016/j.jprot.2008.12.002 19135183

[7] Buchanan BB, Holmgren A, Jacquot J-P, Scheibe R. Fifty years in the thioredoxin field and a bountiful harvest. Biochim Biophys Acta. 2012;1820: 1822–1829. doi: 10.1016/j.bbagen.2012.07.006 22863419

[8] Martin JL. Thioredoxin—a fold for all reasons. Structure. 1995;3: 245–250. doi: 10.1016/s0969-2126(01)00154-x 7788290

[9] Weiss MC, Sousa FL, Mrnjavac N, Neukirchen S, Roettger M, Nelson-Sathi S, et al. The physiology and habitat of the last universal common ancestor. Nat Microbiol. 2016;1: 1–8. doi: 10.1038/nmicrobiol.2016.116 27562259

[10] Meyer Y, Belin C, Delorme-Hinoux V, Reichheld J-P, Riondet C. Thioredoxin and glutaredoxin systems in plants: molecular mechanisms, crosstalks, and functional significance. Antioxid Redox Signal. 2012;17: 1124–1160. doi: 10.1089/ars.2011.4327 22531002

[11] Sengupta R, Holmgren A. The role of thioredoxin in the regulation of cellular processes by S-nitrosylation. Biochim Biophys Acta. 2011 [cited 30 Jan 2012]. doi: 10.1016/j.bbagen.2011.08.012 21878369

[12] Hanschmann E-M, Godoy J, Berndt C, Hudemann C, Lillig CH. Thioredoxins, Glutaredoxins, and Peroxiredoxins-Molecular Mechanisms and Health Significance: From Cofactors to Antioxidants to Redox Signaling. Antioxid Redox Signal. 2013;19: 1539–1605. doi: 10.1089/ars.2012.4599 23397885 PMC3797455

[13] Schürmann P, Buchanan BB. The ferredoxin/thioredoxin system of oxygenic photosynthesis. Antioxid Redox Signal. 2008;10: 1235–1274. doi: 10.1089/ars.2007.1931 18377232

[14] Cha J-Y, Barman DN, Kim MG, Kim W-Y. Stress defense mechanisms of NADPH-dependent thioredoxin reductases (NTRs) in plants. Plant Signal Behav. 2015;10: e1017698. doi: 10.1080/15592324.2015.1017698 26039478 PMC4623241

[15] Rouhier N. Plant glutaredoxins: pivotal players in redox biology and iron–sulphur centre assembly. New Phytol. 2010;186: 365–372. doi: 10.1111/j.1469-8137.2009.03146.x 20074091

[16] Gutsche N, Thurow C, Zachgo S, Gatz C. Plant-specific CC-type glutaredoxins: functions in developmental processes and stress responses. Biol Chem. 2015;396: 495–509. doi: 10.1515/hsz-2014-0300 25781542

[17] Rodríguez-Manzaneque MT, Tamarit J, Bellí G, Ros J, Herrero E. Grx5 is a mitochondrial glutaredoxin required for the activity of iron/sulfur enzymes. Mol Biol Cell. 2002;13: 1109–21. doi: 10.1091/mbc.01-10-0517 11950925 PMC102255

[18] Bandyopadhyay S, Gama F, Molina-Navarro MM, Gualberto JM, Claxton R, Naik SG, et al. Chloroplast monothiol glutaredoxins as scaffold proteins for the assembly and delivery of [2Fe-2S] clusters. EMBO J. 2008;27: 1122–1133. doi: 10.1038/emboj.2008.50 18354500 PMC2323258

[19] Mühlenhoff U, Molik S, Godoy JR, Uzarska MA, Richter N, Seubert A, et al. Cytosolic monothiol glutaredoxins function in intracellular iron sensing and trafficking via their bound iron-sulfur cluster. Cell Metab. 2010;12: 373–385. doi: 10.1016/j.cmet.2010.08.001 20889129 PMC4714545

[20] Liedgens L, Zimmermann J, Wäschenbach L, Geissel F, Laporte H, Gohlke H, et al. Quantitative assessment of the determinant structural differences between redox-active and inactive glutaredoxins. Nat Commun. 2020;11: 1725. doi: 10.1038/s41467-020-15441-3 32265442 PMC7138851

[21] Trnka D, Engelke AD, Gellert M, Moseler A, Hossain MF, Lindenberg TT, et al. Molecular basis for the distinct functions of redox-active and FeS-transfering glutaredoxins. Nat Commun. 2020;11: 3445. doi: 10.1038/s41467-020-17323-0 32651396 PMC7351949

[22] Gallogly MM, Starke DW, Mieyal JJ. Mechanistic and Kinetic Details of Catalysis of Thiol-Disulfide Exchange by Glutaredoxins and Potential Mechanisms of Regulation. Antioxid Redox Signal. 2009;11: 1059–1081. doi: 10.1089/ars.2008.2291 19119916 PMC2842129

[23] Roos G, Foloppe N, Messens J. Understanding the pK(a) of redox cysteines: the key role of hydrogen bonding. Antioxid Redox Signal. 2013;18: 94–127. doi: 10.1089/ars.2012.4521 22746677

[24] Palde PB, Carroll KS. A universal entropy-driven mechanism for thioredoxin-target recognition. Proc Natl Acad Sci U S A. 2015;112: 7960–7965. doi: 10.1073/pnas.1504376112 26080424 PMC4491776

[25] Berndt C, Schwenn J-D, Lillig CH. The specificity of thioredoxins and glutaredoxins is determined by electrostatic and geometric complementarity. Chem Sci. 2015;6: 7049–7058. doi: 10.1039/c5sc01501d 29861944 PMC5947528

[26] Deponte M, Lillig CH. Enzymatic control of cysteinyl thiol switches in proteins. Biol Chem. 2015;396: 401–413. doi: 10.1515/hsz-2014-0280 25581754

[27] Gellert M, Hossain MF, Berens FJF, Bruhn LW, Urbainsky C, Liebscher V, et al. Substrate specificity of thioredoxins and glutaredoxins—towards a functional classification. Heliyon. 2018;5: e02943. doi: 10.1016/j.heliyon.2019.e02943 31890941 PMC6928294

[28] Schwede T, Kopp J, Guex N, Peitsch MC. SWISS-MODEL: An automated protein homology-modeling server. Nucleic Acids Res. 2003;31: 3381–3385. doi: 10.1093/nar/gkg520 12824332 PMC168927

[29] Benkert P, Biasini M, Schwede T. Toward the estimation of the absolute quality of individual protein structure models. Bioinforma Oxf Engl. 2011;27: 343–350. doi: 10.1093/bioinformatics/btq662 21134891 PMC3031035

[30] Biasini M, Bienert S, Waterhouse A, Arnold K, Studer G, Schmidt T, et al. SWISS-MODEL: modelling protein tertiary and quaternary structure using evolutionary information. Nucleic Acids Res. 2014;42: W252–258. doi: 10.1093/nar/gku340 24782522 PMC4086089

[31] Bienert S, Waterhouse A, de Beer TAP, Tauriello G, Studer G, Bordoli L, et al. The SWISS-MODEL Repository-new features and functionality. Nucleic Acids Res. 2017;45: D313–D319. doi: 10.1093/nar/gkw1132 27899672 PMC5210589

[32] Sievers F, Wilm A, Dineen D, Gibson TJ, Karplus K, Li W, et al. Fast, scalable generation of high-quality protein multiple sequence alignments using Clustal Omega. Mol Syst Biol. 2011;7: 539. doi: 10.1038/msb.2011.75 21988835 PMC3261699

[33] Letunic I, Bork P. Interactive Tree Of Life (iTOL) v5: an online tool for phylogenetic tree display and annotation. Nucleic Acids Res. 2021;49: W293–W296. doi: 10.1093/nar/gkab301 33885785 PMC8265157

[34] Dolinsky TJ, Czodrowski P, Li H, Nielsen JE, Jensen JH, Klebe G, et al. PDB2PQR: expanding and upgrading automated preparation of biomolecular structures for molecular simulations. Nucleic Acids Res. 2007;35: W522–525. doi: 10.1093/nar/gkm276 17488841 PMC1933214

[35] Baker NA, Sept D, Joseph S, Holst MJ, McCammon JA. Electrostatics of nanosystems: application to microtubules and the ribosome. Proc Natl Acad Sci U S A. 2001;98: 10037–10041. doi: 10.1073/pnas.181342398 11517324 PMC56910

[36] Humphrey W, Dalke A, Schulten K. VMD: visual molecular dynamics. J Mol Graph. 1996;14: 33–38, 27–28. doi: 10.1016/0263-7855(96)00018-5 8744570

[37] Ling H, Okada K. An efficient Earth Mover’s Distance algorithm for robust histogram comparison. IEEE Trans Pattern Anal Mach Intell. 2007;29: 840–853. doi: 10.1109/TPAMI.2007.1058 17356203

[38] Orchard S, Ammari M, Aranda B, Breuza L, Briganti L, Broackes-Carter F, et al. The MIntAct project—IntAct as a common curation platform for 11 molecular interaction databases. Nucleic Acids Res. 2014;42: D358–63. doi: 10.1093/nar/gkt1115 24234451 PMC3965093

[39] Stark C, Breitkreutz B-J, Reguly T, Boucher L, Breitkreutz A, Tyers M. BioGRID: a general repository for interaction datasets. Nucleic Acids Res. 2006;34: D535–539. doi: 10.1093/nar/gkj109 16381927 PMC1347471

[40] Riondet C, Desouris JP, Montoya JG, Chartier Y, Meyer Y, Reichheld J-P. A dicotyledon-specific glutaredoxin GRXC1 family with dimer-dependent redox regulation is functionally redundant with GRXC2. Plant Cell Environ. 2012;35: 360–373. doi: 10.1111/j.1365-3040.2011.02355.x 21767278

[41] Couturier J, Jacquot J-P, Rouhier N. Toward a refined classification of class I dithiol glutaredoxins from poplar: biochemical basis for the definition of two subclasses. Front Plant Sci. 2013;4: 518. doi: 10.3389/fpls.2013.00518 24385978 PMC3866529

[42] Couturier J, Ströher E, Albetel A-N, Roret T, Muthuramalingam M, Tarrago L, et al. Arabidopsis chloroplastic glutaredoxin C5 as a model to explore molecular determinants for iron-sulfur cluster binding into glutaredoxins. J Biol Chem. 2011;286: 27515–27527. doi: 10.1074/jbc.M111.228726 21632542 PMC3149344

[43] Li S, Lauri A, Ziemann M, Busch A, Bhave M, Zachgo S. Nuclear activity of ROXY1, a glutaredoxin interacting with TGA factors, is required for petal development in Arabidopsis thaliana. Plant Cell. 2009;21: 429–441. doi: 10.1105/tpc.108.064477 19218396 PMC2660636

[44] Murmu J, Bush MJ, DeLong C, Li S, Xu M, Khan M, et al. Arabidopsis basic leucine-zipper transcription factors TGA9 and TGA10 interact with floral glutaredoxins ROXY1 and ROXY2 and are redundantly required for anther development. Plant Physiol. 2010;154: 1492–1504. doi: 10.1104/pp.110.159111 20805327 PMC2971623

[45] Herrera-Vásquez A, Salinas P, Holuigue L. Salicylic acid and reactive oxygen species interplay in the transcriptional control of defense genes expression. Front Plant Sci. 2015;6: 171. doi: 10.3389/fpls.2015.00171 25852720 PMC4365548

[46] Huang L-J, Li N, Thurow C, Wirtz M, Hell R, Gatz C. Ectopically expressed glutaredoxin ROXY19 negatively regulates the detoxification pathway in Arabidopsis thaliana. BMC Plant Biol. 2016;16: 200. doi: 10.1186/s12870-016-0886-1 27624344 PMC5022239

[47] Zander M, Chen S, Imkampe J, Thurow C, Gatz C. Repression of the Arabidopsis thaliana jasmonic acid/ethylene-induced defense pathway by TGA-interacting glutaredoxins depends on their C-terminal ALWL motif. Mol Plant. 2012;5: 831–840. doi: 10.1093/mp/ssr113 22207719

[48] Li N, Muthreich M, Huang L-J, Thurow C, Sun T, Zhang Y, et al. TGACG-BINDING FACTORs (TGAs) and TGA-interacting CC-type glutaredoxins modulate hyponastic growth in Arabidopsis thaliana. New Phytol. 2019;221: 1906–1918. doi: 10.1111/nph.15496 30252136

[49] Ota R, Ohkubo Y, Yamashita Y, Ogawa-Ohnishi M, Matsubayashi Y. Shoot-to-root mobile CEPD-like 2 integrates shoot nitrogen status to systemically regulate nitrate uptake in Arabidopsis. Nat Commun. 2020;11: 641. doi: 10.1038/s41467-020-14440-8 32005881 PMC6994653

[50] Guo D, Song X, Yuan M, Wang Z, Ge W, Wang L, et al. RNA-Seq Profiling Shows Divergent Gene Expression Patterns in Arabidopsis Grown under Different Densities. Front Plant Sci. 2017;8: 2001. doi: 10.3389/fpls.2017.02001 29234331 PMC5712407

[51] Patterson K, Walters LA, Cooper AM, Olvera JG, Rosas MA, Rasmusson AG, et al. Nitrate-Regulated Glutaredoxins Control Arabidopsis Primary Root Growth. Plant Physiol. 2016;170: 989–999. doi: 10.1104/pp.15.01776 26662603 PMC4734588

[52] Ehrary A, Rosas M, Carpinelli S, Davalos O, Cowling C, Fernandez F, et al. Glutaredoxin AtGRXS8 represses transcriptional and developmental responses to nitrate in Arabidopsis thaliana roots. Plant Direct. 2020;4: e00227. doi: 10.1002/pld3.227 32537558 PMC7287413

[53] Zaffagnini M, Bedhomme M, Marchand CH, Couturier JRM, Gao X-H, Rouhier N, et al. Glutaredoxin s12: unique properties for redox signaling. Antioxid Redox Signal. 2012;16: 17–32. doi: 10.1089/ars.2011.3933 21707412

[54] Laporte D, Olate E, Salinas P, Salazar M, Jordana X, Holuigue L. Glutaredoxin GRXS13 plays a key role in protection against photooxidative stress in Arabidopsis. J Exp Bot. 2012;63: 503–515. doi: 10.1093/jxb/err301 21963612 PMC3245481

[55] Rey P, Becuwe N, Tourrette S, Rouhier N. Involvement of Arabidopsis glutaredoxin S14 in the maintenance of chlorophyll content. Plant Cell Environ. 2017;40: 2319–2332. doi: 10.1111/pce.13036 28741719

[56] Moseler A, Aller I, Wagner S, Nietzel T, Przybyla-Toscano J, Mühlenhoff U, et al. The mitochondrial monothiol glutaredoxin S15 is essential for iron-sulfur protein maturation in Arabidopsis thaliana. Proc Natl Acad Sci U S A. 2015;112: 13735–13740. doi: 10.1073/pnas.1510835112 26483494 PMC4640787

[57] Couturier J, Wu H-C, Dhalleine T, Pégeot H, Sudre D, Gualberto JM, et al. Monothiol glutaredoxin-BolA interactions: redox control of Arabidopsis thaliana BolA2 and SufE1. Mol Plant. 2014;7: 187–205. doi: 10.1093/mp/sst156 24203231

[58] Iñigo S, Durand AN, Ritter A, Le Gall S, Termathe M, Klassen R, et al. Glutaredoxin GRXS17 Associates with the Cytosolic Iron-Sulfur Cluster Assembly Pathway. Plant Physiol. 2016;172: 858–873. doi: 10.1104/pp.16.00261 27503603 PMC5047072

[59] Skryhan K, Cuesta-Seijo JA, Nielsen MM, Marri L, Mellor SB, Glaring MA, et al. The Role of Cysteine Residues in Redox Regulation and Protein Stability of Arabidopsis thaliana Starch Synthase 1. PloS One. 2015;10: e0136997. doi: 10.1371/journal.pone.0136997 26367870 PMC4569185

[60] Ojeda V, Nájera VA, González M, Pérez-Ruiz JM, Cejudo FJ. Photosynthetic activity of cotyledons is critical during post-germinative growth and seedling establishment. Plant Signal Behav. 2017;12: e1347244. doi: 10.1080/15592324.2017.1347244 28692378 PMC5640197

[61] Yokochi Y, Fukushi Y, Wakabayashi K, Yoshida K, Hisabori T. Oxidative regulation of chloroplast enzymes by thioredoxin and thioredoxin-like proteins in Arabidopsis thaliana. Proc Natl Acad Sci U S A. 2021;118: e2114952118. doi: 10.1073/pnas.2114952118 34907017 PMC8713810

[62] Kneeshaw S, Gelineau S, Tada Y, Loake GJ, Spoel SH. Selective protein denitrosylation activity of Thioredoxin-h5 modulates plant Immunity. Mol Cell. 2014;56: 153–162. doi: 10.1016/j.molcel.2014.08.003 25201412

[63] Pitzschke A, Djamei A, Teige M, Hirt H. VIP1 response elements mediate mitogen-activated protein kinase 3-induced stress gene expression. Proc Natl Acad Sci U S A. 2009;106: 18414–18419. doi: 10.1073/pnas.0905599106 19820165 PMC2759709

[64] Courteille A, Vesa S, Sanz-Barrio R, Cazalé A-C, Becuwe-Linka N, Farran I, et al. Thioredoxin m4 controls photosynthetic alternative electron pathways in Arabidopsis. Plant Physiol. 2013;161: 508–520. doi: 10.1104/pp.112.207019 23151348 PMC3532281

[65] Daloso DM, Müller K, Obata T, Florian A, Tohge T, Bottcher A, et al. Thioredoxin, a master regulator of the tricarboxylic acid cycle in plant mitochondria. Proc Natl Acad Sci U S A. 2015;112: E1392–1400. doi: 10.1073/pnas.1424840112 25646482 PMC4371975

[66] Arsova B, Hoja U, Wimmelbacher M, Greiner E, Ustün S, Melzer M, et al. Plastidial thioredoxin z interacts with two fructokinase-like proteins in a thiol-dependent manner: evidence for an essential role in chloroplast development in Arabidopsis and Nicotiana benthamiana. Plant Cell. 2010;22: 1498–1515. doi: 10.1105/tpc.109.071001 20511297 PMC2899873

[67] Nekrasov V, Ludwig AA, Jones JDG. CITRX thioredoxin is a putative adaptor protein connecting Cf-9 and the ACIK1 protein kinase during the Cf-9/Avr9- induced defence response. FEBS Lett. 2006;580: 4236–4241. doi: 10.1016/j.febslet.2006.06.077 16831430

[68] Kneeshaw S, Keyani R, Delorme-Hinoux V, Imrie L, Loake GJ, Le Bihan T, et al. Nucleoredoxin guards against oxidative stress by protecting antioxidant enzymes. Proc Natl Acad Sci U S A. 2017. doi: 10.1073/pnas.1703344114 28724723 PMC5547615

[69] Marchal C, Delorme-Hinoux V, Bariat L, Siala W, Belin C, Saez-Vasquez J, et al. NTR/NRX define a new thioredoxin system in the nucleus of Arabidopsis thaliana cells. Mol Plant. 2014;7: 30–44. doi: 10.1093/mp/sst162 24253198

[70] Berndt C, Lillig CH, Wollenberg M, Bill E, Mansilla MC, de Mendoza D, et al. Characterization and Reconstitution of a 4Fe-4S Adenylyl Sulfate/Phosphoadenylyl Sulfate Reductase from Bacillus subtilis. J Biol Chem. 2004;279: 7850–7855. doi: 10.1074/jbc.M309332200 14627706

[71] Chibani K, Tarrago L, Gualberto JM, Wingsle G, Rey P, Jacquot J-P, et al. Atypical thioredoxins in poplar: the glutathione-dependent thioredoxin-like 2.1 supports the activity of target enzymes possessing a single redox active cysteine. Plant Physiol. 2012;159: 592–605. doi: 10.1104/pp.112.197723 22523226 PMC3375927

[72] Serrato AJ, Guilleminot J, Meyer Y, Vignols F. AtCXXS: atypical members of the Arabidopsis thaliana thioredoxin h family with a remarkably high disulfide isomerase activity. Physiol Plant. 2008;133: 611–622. doi: 10.1111/j.1399-3054.2008.01093.x 18384502

[73] Maeda K, Hägglund P, Finnie C, Svensson B, Henriksen A. Structural basis for target protein recognition by the protein disulfide reductase thioredoxin. Struct Lond Engl 1993. 2006;14: 1701–1710. doi: 10.1016/j.str.2006.09.012 17098195

[74] Hossain MF, Bodnar Y, Klein C, Salas CO, Arnér ESJ, Gellert M, et al. Molecular Basis for the Interactions of Human Thioredoxins with Their Respective Reductases. Oxid Med Cell Longev. 2021;2021: 6621292. doi: 10.1155/2021/6621292 34122725 PMC8189816

[75] Yoshida K, Hisabori T. Current Insights into the Redox Regulation Network in Plant Chloroplasts. Plant Cell Physiol. 2023;64: 704–715. doi: 10.1093/pcp/pcad049 37225393 PMC10351500

[76] Gutsche N, Holtmannspötter M, Maß L, O’Donoghue M, Busch A, Lauri A, et al. Conserved redox-dependent DNA binding of ROXY glutaredoxins with TGA transcription factors. Plant Direct. 2017;1: e00030. doi: 10.1002/pld3.30 31245678 PMC6508501

[77] Nietzel T, Mostertz J, Ruberti C, Née G, Fuchs P, Wagner S, et al. Redox-mediated kick-start of mitochondrial energy metabolism drives resource-efficient seed germination. Proc Natl Acad Sci U S A. 2020;117: 741–751. doi: 10.1073/pnas.1910501117 31871212 PMC6955286

[78] Spínola MC, Pérez-Ruiz JM, Pulido P, Kirchsteiger K, Guinea M, González M, et al. NTRC new ways of using NADPH in the chloroplast. Physiol Plant. 2008;133: 516–524. doi: 10.1111/j.1399-3054.2008.01088.x 18346073

[79] Juniar L, Tanaka H, Yoshida K, Hisabori T, Kurisu G. Structural basis for thioredoxin isoform-based fine-tuning of ferredoxin-thioredoxin reductase activity. Protein Sci Publ Protein Soc. 2020;29: 2538–2545. doi: 10.1002/pro.3964 33015914 PMC7679956

[80] Dai S, Saarinen M, Ramaswamy S, Meyer Y, Jacquot JP, Eklund H. Crystal structure of Arabidopsis thaliana NADPH dependent thioredoxin reductase at 2.5 A resolution. J Mol Biol. 1996;264: 1044–1057. doi: 10.1006/jmbi.1996.0695 9000629

[81] Kirkensgaard KG, Hägglund P, Finnie C, Svensson B, Henriksen A. Structure of Hordeum vulgare NADPH-dependent thioredoxin reductase 2. Unwinding the reaction mechanism. Acta Crystallogr D Biol Crystallogr. 2009;65: 932–941. doi: 10.1107/S0907444909021817 19690371 PMC2733882

[82] Lennon BW, Williams CH, Ludwig ML. Twists in catalysis: alternating conformations of Escherichia coli thioredoxin reductase. Science. 2000;289: 1190–1194. doi: 10.1126/science.289.5482.1190 10947986

[83] Fritz-Wolf K, Kehr S, Stumpf M, Rahlfs S, Becker K. Crystal structure of the human thioredoxin reductase-thioredoxin complex. Nat Commun. 2011;2: 383. doi: 10.1038/ncomms1382 21750537

[84] Yoshida K, Hisabori T. Distinct electron transfer from ferredoxin–thioredoxin reductase to multiple thioredoxin isoforms in chloroplasts. Biochem J. 2017;474: 1347–1360. doi: 10.1042/BCJ20161089 28246333

