# Supplementary material for: Breakdown of Arabidopsis thaliana thioredoxins and glutaredoxins based on electrostatic similarity–Leads to common and unique interaction partners and functions
Source: PLoS One. 2023 Sep 11;18(9):e0291272. doi: 10.1371/journal.pone.0291272 (PMC10495010; doi:10.1371/journal.pone.0291272)
Supplement: S1 File — (PDF) [file pone.0291272.s002.pdf]

Supplementary material to:

Breakdown of *Arabidopsis thaliana* thioredoxins and glutaredoxins based on electrostatic similarity – leads to common and unique interaction partners and functions

Bodnar, Yana[1,2]; Gellert, Manuela[1]; Hossain, Faruq Mohammed[1,3]; and Lillig, Christopher Horst[1]*

From the Institute for Medical Biochemistry and Molecular Biology, University Medicine Greifswald, Germany (1) and Institute for Physics, University of Greifswald, Germany (2)

* address for correspondence: Christopher Horst Lillig, Institute for Medical Biochemistry and Molecular Biology, University Medicine Greifswald, Ferdinand-Sauerbruch-Straße. DE-17475 Greifswald, Germany, Tel: +49 3834 865407, Fax: +49 3834 865402, horst@lillig.de

[3] present address: Interfaculty Institute for Genetics and Functional Genomics, University Medicine Greifswald, Germany

Table of Contents

Supplementary Figure 1..2
Supplementary Table 1..3
References..5

Additional file: Supplementary spreadsheet listing the potential interaction partners of all *Arabidopsis thaliana* redoxins extracted from the literature and databases (see main text).

Supplementary Figure 1

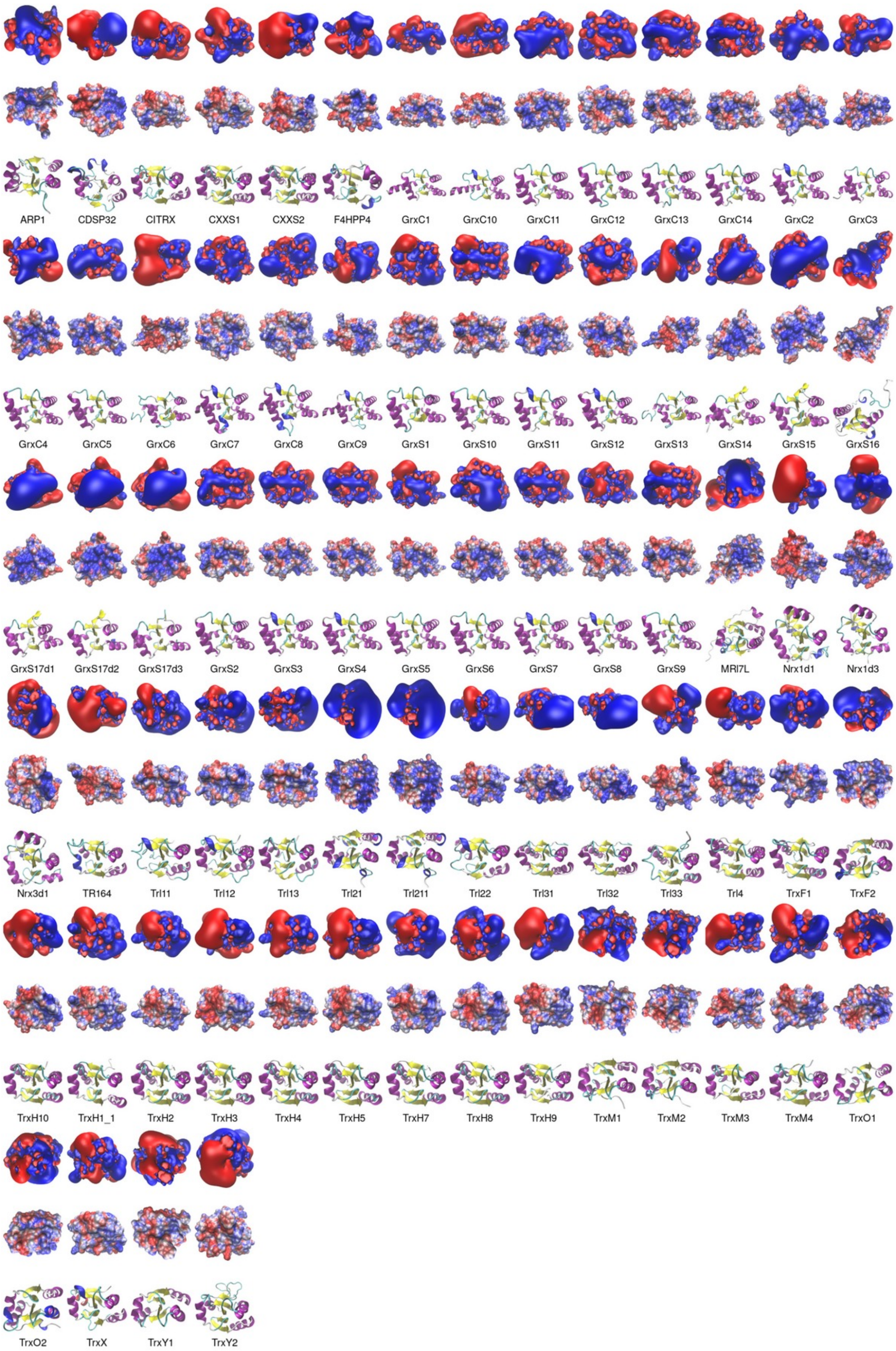

Supplementary Figure 1 – Electrostatic features of the active site contact areas of the redoxins from *Arabidopsis thaliana*. The first rows depict the electrostatic potential isosurfaces at +/- 1 $k \cdot T \cdot e^{-1}$. The second row depicts the electrostatic potential at +/- 4 $k \cdot T \cdot e^{-1}$ mapped to the water-accessible surface of the proteins. Blue: positive, red negative potential. The third row depicts the proteins in cartoon models, helices are colored in purple, sheets in yellow. The proteins were arranged with the N-terminal active site thiol in the middle of the models. The electrostatic similarity of the whole proteins was computed as outlined in the methods section, see reference [1].

Supplementary Table 1

3D structures analyzed in this work. When available, an experimental 3D structure was used for analysis. For the structures generated by homology modeling using the Swiss model server [2], both template structure form the PDB and the quality parameter QMEAN [3] are listed.

protein	active site	PDB entry	template	QMEAN
***Arabidopsis thaliana* glutaredoxins**				
GrxC1	CGYC		2e7p.1.C	0.9
GrxC2	CPFC		5kqa.1.A	0.79
GrxC3	CPFC		2jad.1.A	-1.06
GrxC4	CPFC		3fz9.1.A	-0.4
GrxC5	CSYC/S	3rhb, 3rhc		
GrxC6	CC		2mxn.1.A	-2.11
GrxC7	CC		5gtx.1.A	-0.7
GrxC8	CC		5gtx.1.A	-0.07
GrxC9	CC		5gtx.1.A	-0.85
GrxC10	CC		5gtx.1.A	-0.33
GrxC11	CC		2e7p.1.A	-0.38
GrxC12	CC		3rhb.1.A	0.25
GrxC13	CC		3fz9.1.A	-1.25
GrxC14	CC		3fz9.1.A	-0.86
GrxS1	CC		1z7p.1.A	-1.73
GrxS2	CC		3fz9.1.A	-0.25
GrxS3	CC		5kqa.1.A	-0.37
GrxS4	CC		5kqa.1.A	-0.13
GrxS5	CC		5gtx.1.A	0.12
GrxS6	CC		5gtx.1.A	-0.32
GrxS7	CC		5kqa.1.A	-0.33

GrxS8	CC		5kqa.1.A	-0.42
GrxS9	CC		3fz9.1.A	-0.21
GrxS10	CC		3fz9.1.A	0.53
GrxS11	CC		5gtx.1.A	0.25
GrxS12	CC		3fz9.1.A	0.85
GrxS13	CC		2mxn.1.A	-1.92
GrxS14	CGFS	3ipz, 2lku, 2mma		
GrxS15	CGFS		2mxn.1.A	-1.72
GrxS16	CGFS	2lwf		
GrxS17 d1	CGFS		2wci.1.A	0.32
GrxS17 d2	CGFS		2yan.1.A	0.23
GrxS17 d3	CGFS		2mxn.1.A	0.4
Arabidopsis thaliana **thioredoxins**				
Trxf1	CGPC		1faa.1.A	-0.54
Trxf2	CGPC	7c2b		
Trxh1	CGPC	1xfl		
Trxh2	CGPC		2vm1.1.A	0.19
Trxh3	CPPC		2iwt.1.A	-0.2
Trxh4	CPPC		2vm1.1.A	0.66
Trxh5	CPPC		2iwt.1.A	-0.22
Trxh7	CGPC		2vm1.1.A	-1.16
Trxh8	CGPC		2vm1.1.A	-0.5
Trxh9	CGPC		3d22.1.A	0.36
Trxh10	CVPC		3d21.1.A	0.83
Trxm1	CGPC	7c65		
Trxm2	CGPC	7c3f		
Trxm3	CGPC		1dby.1.A	-1.35
Trxm4	CGPC		1fb0.1.A	1.37
Trxo1	CGPC	6g61	2vm1.1.A	0.09
Trxo2	CGPC	7bzk	2vm1.1.A	-0.49
Trxx	CGPC		2l4q.1.A	-1.21
Trxy1	CGPC		3dxb.1.A	-0.07

Trxy2	CGPC		3p2a.1.A	-1.06
ClTRX	CGPC		2l4q.1.A	-1.94
Nrx1_d1	CGPC		1oc8.1.A	-1.36
Nrx1_d3	CPPC		1fg4.1.A	-1.74
Nrx3_d1	CRPC		1o73.1.A	-2.25
F4HPP4 / TARWCGPC	CGPC		6ap5.1.A	-3.24
TR164 / HCF164	CEVC		3dxb.1.B	-1.81
Trl11 / Lilium1	CGGC		2b5e.1.A	-4.25
Trl22 / Lilium2	CASC		2b5e.1.A	-2.9
Trl12 / Lilium3	CGGC		2b5e.1.A	-1.86
Trl13 / Lilium4	CGGC		5gu6.1.A	-1.6
Trl21 / Lilium5	CGSC	6lyx, 6lyw		
Trl4 / Lilium6	CGSC		5g31.1.A	-1.29
Trl33	CGVC		3d22.1.A	-1.88
CXXS1	CIPS		3d22.1.A	-0.4
CXXS2	CLPS		3d22.1.A	-0.53
Trl31 / WCRK1	CRKC		5nyk.1.A	-0.34
Trl32 / WCRK2	CRKC		5nyk.1.A	0.04
CDSP32	CGPC		4ij3.1.A	-3.64
***Arabidopsis thaliana* thioredoxin reductases**				
FTR		7c2b		
NTR1			1vdc.1.A	-0.94
NTR2		1vdc		
NTRC			2pu9.1.A	-0.9

References

1. Gellert M, Hossain MF, Berens FJF, Bruhn LW, Urbainsky C, Liebscher V, et al. Substrate specificity of thioredoxins and glutaredoxins - towards a functional classification. Heliyon. 2019;5: e02943. doi:10.1016/j.heliyon.2019.e02943

2. Schwede T, Kopp J, Guex N, Peitsch MC. SWISS-MODEL: An automated protein homology-modeling server. Nucleic Acids Res. 2003;31: 3381–3385.

3. Benkert P, Biasini M, Schwede T. Toward the estimation of the absolute quality of individual protein structure models. Bioinforma Oxf Engl. 2011;27: 343–350. doi:10.1093/bioinformatics/btq662
